# Safety of primary nasotracheal intubation in the pediatric intensive care unit (PICU)

**DOI:** 10.1007/s44253-024-00035-4

**Published:** 2024-02-23

**Authors:** Laurence Ducharme-Crevier, Jamie Furlong-Dillard, Philipp Jung, Fabrizio Chiusolo, Matthew P. Malone, Shashikanth Ambati, Simon J. Parsons, Conrad Krawiec, Awni Al-Subu, Lee A. Polikoff, Natalie Napolitano, Keiko M. Tarquinio, Asha Shenoi, Andrea Talukdar, Palen P. Mallory, John S. Giuliano, Ryan K. Breuer, Krista Kierys, Serena P. Kelly, Makoto Motomura, Ron C. Sanders, Ashley Freeman, Yuki Nagai, Lily B. Glater-Welt, Joseph Wilson, Mervin Loi, Michelle Adu-Darko, Justine Shults, Vinay Nadkarni, Guillaume Emeriaud, Akira Nishisaki, for the National Emergency Airway Registry for Children (NEAR4KIDS), Pediatric Acute Lung Injury, Sepsis Investigators (PALISI) Network

**Affiliations:** 1 Pediatric Intensive Care Unit, Department of Pediatrics, CHU Sainte-Justine Université de Montréal, Montréal, QC H3T 1C5 Canada; 2 Department of Pediatric Critical Care, Norton Children’s Hospital, University of Louisville, Louisville, KY USA; 3 Department of Pediatrics, University Hospital Schleswig Holstein, Campus Luebeck, Luebeck, Germany; 4 Department of Anesthesia and Critical Care, IRCCS Bambino Gesù Children’s Hospital, Rome, Italy; 5 Division of Critical Care Medicine, Department of Pediatrics, The University of Arkansas for Medical Sciences, Arkansas Children’s Hospital, Little Rock, AR USA; 6 Division of Pediatric Critical Care, Department of Pediatrics, Albany Medical Center, Albany, NY USA; 7 Section of Critical Care Medicine, Department of Pediatrics, Alberta Children’s Hospital, Calgary, AB Canada; 8 Pediatric Critical Care, Department of Pediatrics, College of Medicine, Penn State Health Children’s Hospital, Hershey, PA USA; 9 Department of Pediatrics, School of Medicine and Public Health, University of Wisconsin, Madison, WI USA; 10 Division of Pediatric Critical Care Medicine, Warren Alpert Medical School of Brown University, Providence, RI USA; 11 Respiratory Therapy Department, Children’s Hospital of Philadelphia, Philadelphia, PA USA; 12 College of Health Professions, the Medical University of South Carolina, Charleston, SC USA; 13 Division of Pediatric Critical Care, Department of Pediatrics, University of Kentucky School of Medicine, Lexington, KY USA; 14 Pediatric Critical Care, Medical Center/Children’s Hospital and Medical Center of Omaha, University of Nebraska, Omaha, NE USA; 15 Division of Pediatric Critical Care Medicine, Duke University, Durham, NC USA; 16 Department of Pediatrics (Critical Care Medicine), Yale University School of Medicine, New Haven, CT USA; 17 Division of Critical Care Medicine, Department of Pediatrics, Oishei Children’s Hospital, Buffalo, NY USA; 18 Pediatric Intensive Care Unit, Penn State Health, Philadelphia, PA USA; 19 Division of Pediatric Critical Care, OHSU Doernbecher Children’s Hospital, Portland, OR USA; 20 Division of Pediatric Critical Care Medicine, Aichi Children’s Health and Medical Center, Obu, Aichi Japan; 21 Section of Critical Care, Department of Pediatrics, UAMS/Arkansas Children’s Hospital, Little Rock, AR USA; 22 Pediatric Critical Care, Department of Pediatrics, Children’s Hospital of Georgia at the Medical College of Georgia, Augusta, GA USA; 23 Division of Pediatric Critical Care Medicine, Kobe Children’s Hospital, Kobe, Hyogo Japan; 24 Pediatric Critical Care Medicine, Cohen Children’s Medical Center of New York/Northwell, Queens, NY USA; 25 Pediatric Critical Care Medicine, University of Louisville, Louisville, KY USA; 26 Department of Pediatric Subspecialties, Children’s Intensive Care Unit KK Women’s and Children’s Hospital, Singapore, Singapore; 27 Division of Pediatric Critical Care, Department of Pediatrics, University of Virginia Hospital, Charlottesville, VA USA; 28 Department of Biostatistics, Epidemiology, and Informatics, Perelman School of Medicine, University of Pennsylvania, Philadelphia, PA USA; 29 Department of Anesthesiology and Critical Care Medicine, The Children’s Hospital of Philadelphia, Philadelphia, PA USA

**Keywords:** Nasal intubation, Endotracheal intubation, Adverse events, Pediatric intensive care

## Abstract

**Background:**

Nasal tracheal intubation (TI) represents a minority of all TI in the pediatric intensive care unit (PICU). The risks and benefits of nasal TI are not well quantified. As such, safety and descriptive data regarding this practice are warranted.

**Methods:**

We evaluated the association between TI route and safety outcomes in a prospectively collected quality improvement database (National Emergency Airway Registry for Children: NEAR4KIDS) from 2013 to 2020. The primary outcome was severe desaturation (SpO_2_ > 20% from baseline) and/or severe adverse TI-associated events (TIAEs), using NEAR4KIDS definitions. To balance patient, provider, and practice covariates, we utilized propensity score (PS) matching to compare the outcomes of nasal vs. oral TI.

**Results:**

A total of 22,741 TIs [nasal 870 (3.8%), oral 21,871 (96.2%)] were reported from 60 PICUs. Infants were represented in higher proportion in the nasal TI than the oral TI (75.9%, vs 46.2%), as well as children with cardiac conditions (46.9% vs. 14.4%), both *p* < 0.001. Severe desaturation or severe TIAE occurred in 23.7% of nasal and 22.5% of oral TI (non-adjusted *p* = 0.408). With PS matching, the prevalence of severe desaturation and or severe adverse TIAEs was 23.6% of nasal vs. 19.8% of oral TI (absolute difference 3.8%, 95% confidence interval (CI): − 0.07, 7.7%), *p* = 0.055. First attempt success rate was 72.1% of nasal TI versus 69.2% of oral TI, *p* = 0.072. With PS matching, the success rate was not different between two groups (nasal 72.2% vs. oral 71.5%, *p* = 0.759).

**Conclusion:**

In this large international prospective cohort study, the risk of severe peri-intubation complications was not significantly higher. Nasal TI is used in a minority of TI in PICUs, with substantial differences in patient, provider, and practice compared to oral TI.

A prospective multicenter trial may be warranted to address the potential selection bias and to confirm the safety of nasal TI.

## Introduction

Many patients admitted to the pediatric intensive care unit (PICU) require tracheal intubation (TI) and mechanical ventilation to support the airway or a failing respiratory system, minimize the work of breathing or the systemic oxygen consumption, control the ventilatory drive, and/or support a failing heart. Intubation is a lifesaving maneuver that has inherent risks [1–4]. Indeed, TI-associated events (TIAEs) were reported in up to 20% of intubation attempts [4]. Tracheal intubation can be performed by the oral or the nasal route. Nasal TI involves passing an endotracheal tube through the naris and into the nasopharynx and the trachea and typically requires some manipulation with forceps.

The National Emergency Airway Registry for Children (NEAR4KIDS) is an international collaborative quality improvement (QI) initiative and registry of TI from PICUs and emergency departments. From this registry, oral intubation represented 95.8% of all TI [4]. The choice of the oral versus nasal route for TI is usually determined by the physician’s own experience and the clinical context. Each route has its advantages and disadvantages [5–7]. Limited evidence exists for the safety of nasal TI, although the oral route is recommended for rapid sequence intubation [6]. It is assumed that oral TI allows more expeditious management of the airway in emergent situations, and it may therefore cause less TIAEs [7]. Nasal TI is however associated with lower rate of unplanned extubations [8] and may increase comfort [6, 9]. Data regarding nasal TI in the PICU is scarce. It is therefore important to assess its safety, to provide knowledge of its related risks. To address this knowledge gap, we utilized the NEAR4KIDS database with the aim to assess the use of nasal TI in multiple PICUs. We hypothesized that patients receiving primary nasal TI have higher risk of severe peri-intubation-related events, desaturation (SpO_2_ decline > 20% from baseline), and/or severe TIAEs, compared to those receiving oral TI.

## Materials and methods

The NEAR4KIDS registry is a quality improvement initiative comprised of prospectively collected TI data from 60 international PICUs. This registry collaborative was approved by the Institutional Review Board (IRB) at the Data Coordinating Center under the study title “Observation of Multi-center Quality Improvement Project: Improving Safety and Quality of Tracheal Intubation Practice in Pediatric ICUs” (Children’s Hospital of Philadelphia IRB 09–007253). IRB approval or exemption was obtained at each participating site. Data collected for each TI event included patient characteristics (age, primary diagnosis, indication for TI, history or features suggestive of a difficult airway), provider (discipline and training level), TI characteristics (route of intubation, equipment, medications used), and TI clinical outcomes. Procedures were followed in accordance with the ethical standards of the responsible committee on human experimentation (institutional or regional) and with the Helsinki Declaration of 1975. Each center follows a data compliance plan to ensure at least 95% of all site TIs are captured with high data accuracy [2, 4].

### Inclusion and exclusion criteria

In this study, we included primary TI for children < 18 years of age in the PICU and pediatric cardiac intensive care unit (CICU) from January 2013 to December 2020. Intubations in the operating room, in the ED, or out-of-hospital location were excluded [10]. Exchange of an existing endotracheal tube was also excluded.

### Exposure and outcome measures

The primary exposure variable was initial nasal TI, defined as the first route reported on the first attempt. Our primary outcome was a composite of rate for peri-intubation severe adverse events: severe oxygen desaturation and/or severe TIAEs. Severe desaturation was defined as pulse oximetry saturation (SpO_2_) decline more than 20% from pre-procedure baseline during the first TI attempt [11, 12]. Severe TIAEs, by NEAR4KIDS definition, included cardiac arrest, esophageal intubation with delayed recognition, emesis with witnessed aspiration, hypotension requiring intervention (intravenous fluid and/or vasopressors), laryngospasm, pneumothorax/pneumomediastinum, or direct airway injury. These events must occur within 20 min of the TI attempt in order to meet the operational definition of TIAEs. The definition of TIAEs was described in the shared NEAR4KIDS operational definition documents, and each site PI and data coordinator received the training by the Data Coordinating Center. Additional details are available in a prior publication [3].

Our secondary outcomes included the overall TIAE rate (minor and severe) and the number of TI attempts. Minor TIAEs included mainstem bronchial intubation, esophageal intubation with immediate recognition, emesis without aspiration, hypertension requiring therapy, epistaxis, dental or lip trauma, medication error, arrhythmia, or pain and/or agitation requiring additional medication and causing delay in TI. The data were entered into secure Research Electronic Data Capture (REDCap®) system hosted by the Data Coordinating Center [13].

### Statistical analysis

#### Sample size calculation

The minimal sample size and statistical power were estimated a priori. To detect an absolute difference of 4% in the primary outcome (severe oxygen desaturation and/or severe TIAEs), with an estimated incidence of 14% of severe TIAEs related to nasal TI in the NEAR4KIDS registry, a sample size of 14,489 TIs (with a proportion of 4% of nasal TI) was necessary to achieve a power of 80%.

Summary statistics were provided as percentages for categorical variables and either median with interquartile range (*IQR*, 25th–75th percentile) or mean and standard deviation (SD) for continuous variables. Categorical variables were compared between groups using the chi-square test, whereas continuous variables were compared using the Wilcoxon rank-sum test. Univariable and multivariable logistic regressions were performed to evaluate the association between nasal TI and the primary composite outcome (rate of severe oxygen desaturation > 20% from baseline and/or severe TIAEs). In the multivariate model, variables that were chosen a priori were patient age and diagnostic category, TI for indications of respiratory and shock, and provider level of training. In addition, the following variables were added as they were unbalanced at baseline and potential confounders: device (video laryngoscope vs. direct laryngoscopy), history of difficult airway, vagolytic and paralytic use, and apneic oxygenation utilization. To further address the imbalance in patient, provider, and practice characteristics, we performed a propensity score (PS) analysis, with 1:1 matching without replacement. The PS was calculated for each patient as the predicted probability of nasal TI. With the calculated PS, nearest one-to-one neighbor matching without replacement was performed with a caliper width no greater than 0.2 times the SD of the logit of the PS to generate matched cohorts in which covariates are balanced. After confirming that we had achieved acceptable balance in the covariates, the association between the exposure and the primary outcome was assessed using the matched cohort.

## Results

### Nasotracheal intubation

From a total of 25,363 encounters reported in the NEAR4KIDS cohort during the study period, 2622 encounters were excluded based upon exclusion criteria. We included 22,741 TI, 870 (3.8%) nasal TI, and 21,871 (96.2%) oral TI, from 60 PICUs (Table 1). Infants and patients with cardiac conditions more often underwent nasal TI than other demographic groups (*p* < 0.001). Nasal TI was used more commonly for procedural indication and less commonly for oxygenation or ventilation failure indication (*p* < 0.001). Nasal TI was used less frequently for the patients with a difficult airway history and by fellow (as compared to attending) physicians.

**Table 1:** Patient, provider, and practice characteristics stratified by route of intubation (*N* = 22,741)

Characteristics	Nasal TI (*n* = 870)	Oral TI (*n* = 21,871)	*p*-value
**Patient**			
**Patient age**			< 0.001
Infant	660 (75.9%)	10,099 (46.2%)	
Young child (1–7 years)	159 (18.3%)	6985 (31.9%)	
Child (8–17 years)	51 (5.8%)	4787 (21.9%)	
**Sex**			0.076
Male	509 (58.6%)	12,136 (55.5%)	
Female	360 (41.4%)	9728 (44.5%)	
Weight (median, IQR)	4.3 (3.3–8.6)	9.8 (5.0–21.1)	< 0.001
PIM2%≠ (median, IQR)	3.0 (1.1–9.3)	2.1 (0.8–5.4)	< 0.001
**Diagnostic category**			< 0.001
Respiratory	206 (23.7%)	10,150 (46.4%)	
Cardiac	408 (46.9%)	3153 (14.4%)	
Neurological	96 (11.0%)	3906 (17.9%)	
Shock	34 (3.9%)	1885 (8.6%)	
Trauma/TBI	9 (1.0%)	519 (2.4%)	
Other	117 (13.4%)	2258 (10.3%)	
**Indication for intubation**			
Oxygenation failure	181 (20.8%)	7848 (35.9%)	< 0.001
Ventilation failure	229 (26.3%)	8064 (36.9%)	< 0.001
Upper airway obstruction	53 (6.1%)	2129 (9.7%)	< 0.001
Neurological	47 (5.4%)	1893 (8.7%)	0.001
Procedural	246 (28.3%)	3468 (15.9%)	< 0.001
Shock	58 (6.7%)	2796 (12.8%)	< 0.001
**Difficult airway**			
History of difficult airway	65 (7.5%)	2930 (13.4%)	< 0.001
Difficult airway feature	336 (38.6%)	6322 (28.9%)	< 0.001
**Provider**			< 0.001
Attending	301 (34.6%)	3964 (18.1%)	
Fellow	250 (28.8%)	10,434 (47.8%)	
Resident	101 (11.6%)	2556 (11.7%)	
Nurse practitioner	65 (7.5%)	2137 (9.8%)	
Hospitalist	7 (0.8%)	127 (0.6%)	
Respiratory therapist	0 (0.0%)	208 (1.0%)	
Subspecialist — other	145 (16.7%)	2426 (11.1%)	
**Practice**			
Atropine	175 (20.1%)	5455 (24.9%)	0.001
Ketamine	432 (49.7%)	7271 (33.2%)	< 0.001
Paralysis	685 (78.7%)	19,567 (89.5%)	< 0.001
Video laryngoscopy	116 (13.3%)	7134 (32.6%)	< 0.001

≠ PIM2 denotes Pediatric Index of Mortality 2. PIM 2 was only available in 12,472 encounters

*IQR *Interquartile range, *TBI *Traumatic brain injury

### Primary outcome

In the univariate analysis, the primary outcome (occurrence of either severe desaturation and/or severe TIAEs) was reported in 23.7% of nasal TI and 22.5% of oral TIs (*p* = 0.408) (Table 2). Severe desaturation (SpO_2_ decline > 20% from baseline) occurred in 22.4% of nasal TI vs. 19.2% of oral TI (*p* = 0.312). Severe TIAEs were reported in 2.2% of nasal TI vs. 5.6% of oral TI (*p* < 0.001).

**Table 2:** Adverse tracheal intubation-associated events (TIAEs) and desaturation by intubation route (univariable analysis)

Adverse event	Nasal TI (*n* = 870)	Oral TI (*n* = 21,871)	*p*-value
**Severe TIAE or/and severe desaturation**	206 (23.7%)	4917 (22.5%)	0.408
**Any TIAE**	74 (8.5%)	3036 (13.9%)	< 0.001
**Severe TIAE (all)**	19 (2.2%)	1224 (5.6%)	< 0.001
Cardiac arrest with ROSC	5 (0.6%)	240 (1.1%)	
Cardiac arrest without ROSC	1 (0.1%)	35 (0.2%)	
Esophageal intubation with delayed recognition	4 (0.5%)	74 (0.3%)	
Emesis with aspiration	1 (0.1%)	123 (0.6%)	
Hypotension requiring intervention	6 (0.7%)	666 (3.1%)	
Laryngospasm	0 (0.0%)	51 (0.2%)	
Dental injury	2 (0.2%)	80 (0.4%)	
Pneumothorax/pneumomediastinum	1 (0.1%)	31 (0.1%)	
Airway injury	0 (0.0%)	16 (0.1%)	
**Non-severe TIAE (all)**	57 (6.6%)	2071 (9.5%)	0.003
Esophageal intubation with immediate recognition	33 (3.8%)	1015 (4.6%)	
Dysrhythmia	5 (0.6%)	250 (1.1%)	
Mainstem bronchial intubation	2 (0.2%)	621 (2.8%)	
Emesis without aspiration	2 (0.2%)	139 (0.6%)	
Pain and agitation delaying the process	1 (0.1%)	39 (0.2%)	
Lip injury	3 (0.3%)	123 (0.6%)	
Epistaxis	11 (1.3%)	15 (0.1%)	
Hypertension requiring intervention	1 (0.1%)	24 (0.1%)	
Medication error	0 (0.0%)	8 (0.0%)	
**Severe desaturation** (SpO_2_ decline > 20%)	195 (22.4%)	4192 (19.2%)	0.020

*TI *Tracheal intubation, *TIAE *Tracheal intubation-associated event, *ROSC *Return of spontaneous circulation

However, multivariable logistic regression did not show higher likelihood of severe oxygen desaturation and/or severe TIAEs with nasal TI route (*OR* 1.03, 95% *CI*: 0.87–1.22, *p* = 0.704) (Table 3). One-to-one PS matching was possible for 869 patients with nasal TI. The covariates were well balanced between two groups (Table 4).

**Table 3:** Multivariable logistic regression analysis of the association between the route of tracheal intubation (TI) and severe desaturation and/or severe tracheal intubation adverse events (TIAEs)

Patient, provider, practice factors	Adjusted odds ratio (95% *CI*)	*p*-value
**Route of intubation**		
Oral	Reference	
Nasal	1.03 (0.87–1.22)	0.704
**Age**		
Infant (< 1 year)	Reference	
Young child (1–7 year)	0.90 (0.83–0.97)	0.008
Older child (8–17 year)	0.72 (0.65–0.79)	< 0.001
**Diagnostic category**		
Respiratory	Reference	
Cardiac	1.20 (1.08–1.32)	< 0.001
Neurological	0.55 (0.48–0.62)	< 0.001
Shock	1.02 (0.90–1.16)	0.769
Trauma/TBI	0.80 (0.63–1.01)	0.060
Other	1.10 (0.98–1.23)	0.112
**Indication for intubation**		
Oxygenation failure	1.64 (1.53–1.76)	< 0.001
Ventilation failure	1.12 (1.04–1.20)	0.002
Upper airway obstruction	0.97 (0.87–1.08)	0.599
Neurological	0.93 (0.81–1.06)	0.289
Procedural	0.73 (0.65–0.81)	< 0.001
Shock	1.34 (1.21–1.49)	< 0.001
**Difficult airway**		
Difficult airway history	1.32 (1.21–1.45)	< 0.001
Difficult airway feature	1.40 (1.30–1.50)	< 0.001
**Provider**		
Attending	Reference	
Fellow	0.93 (0.85–1.01)	0.092
Resident	1.35 (1.20–1.52)	< 0.001
Nurse practitioner	1.14 (1.01–1.30)	0.050
Hospitalist	0.84 (0.51–1.38)	0.495
Respiratory therapist	1.29 (0.93–1.79)	0.128
Subspecialists/other	1.08 (0.96–1.21)	0.224
**Medication**		
Atropine	1.17 (1.09–1.26)	< 0.001
Ketamine	1.01 (0.94–1.08)	0.871
Neuromuscular blockade	0.92 (0.83–1.02)	0.119
Video laryngoscopy	0.99 (0.92–1.06)	0.740
Apneic oxygenation	0.99 (0.90–1.08)	0.802

*CI *Confidence interval, *TBI *Traumatic brain injury, *TI *Tracheal intubation

**Table 4:** Characteristics and primary outcome of nasal TI and oral TI groups after 1:1 propensity score matching without replacement

Variable name	Nasal TI (*n* = 869)	Oral TI (*n* = 869)	**Standardized absolute mean difference (SAMD)#**
**Age category**			
Infant	659 (75.8%)	659 (75.8%)	0%
Young child (1–7 years)	159 (18.3%)	148 (17%)	3.3%
Older child (8–17 years)	51 (5.9%)	62 (7.1%)	5.1%
**Diagnostic category**			
Respiratory	6 (23.7%)	240 (27.6%)	9%
Cardiac	408 (47.0%)	426 (49.0%)	4.1%
Neurological	96 (11.0%)	95 (10.9%)	0.4%
Shock	34 (3.9%)	28 (3.2%)	3.7%
Trauma/TBI	9 (1.0%)	10 (1.2%)	1.1%
**Indication**			
Oxygenation failure	181 (20.8%)	187 (21.5%)	1.7%
Ventilation failure	229 (26.4%)	222 (25.5%)	1.8%
Upper airway obstruction	53 (6.1%)	44 (5.1%)	4.5%
Neurological failure	47 (5.4%)	50 (5.8%)	1.5%
Procedural	245 (28.2%)	221 (25.4%)	6.2%
Shock	58 (6.7%)	65 (7.5%)	3.1%
**Difficult airway**			
Difficult airway history	65 (7.5%)	62 (7.1%)	1.3%
Difficult airway clinical feature	336 (38.7%)	324 (37.3%)	2.8%
**Provider**			
Attending	301 (34.6%)	301 (34.6%)	0.0%
Fellow	250 (28.8%)	253 (29.1%)	0.8%
Resident	101 (11.6%)	87 (10.0%)	5.2%
Nurse practitioner	65 (7.5%)	61 (7.0%)	1.8%
Respiratory therapist	0 (0%)	10 (1.2%)	15.3%
Hospitalist	7 (0.8%)	2 (0.2%)	8.0%
Subspecialist/other	145 (16.7%)	155 (17.8%)	3.0%
**Medication**			
Atropine	175 (20.1%)	178 (20.5%)	0.9%
Ketamine	432 (49.7%)	425 (48.9%)	1.6%
Paralysis	684 (78.7%)	673 (77.4%)	3.1%
Video laryngoscopy	116 (13.3%)	111 (12.8%)	1.7%
Apneic oxygenation	42 (4.8%)	47 (5.4%)	2.6%
**Overall average SAMD**	N/A	N/A	**3.3%**

*TBI *Traumatic brain injury, *TI *Tracheal intubation, *TIAE *Tracheal intubation-associated events. *N/A*, not applicable

Standardized absolute mean difference (SAMD)#

This value is calculated as follows: *SAMD* (the absolute value of the difference in average outcome between cases and controls, divided by the square root of the average of the sample variance for cases and controls) × 100

**Fig. 1:**
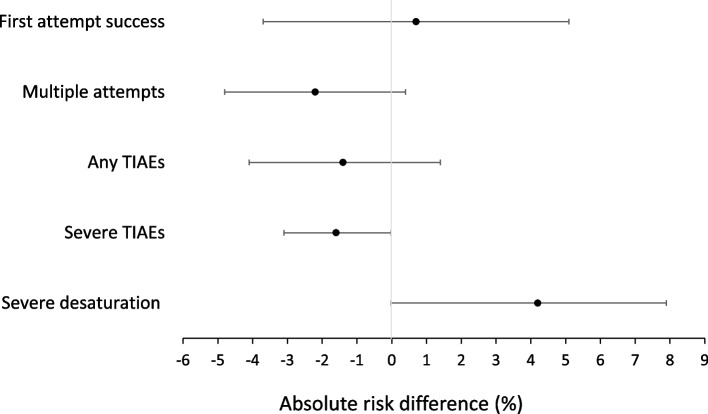
Absolute risk difference (%), 95% confidence interval

In the PS-matched analyses, nasal TI was not associated with the composite primary outcome of severe desaturation and/or severe TIAE (nasal 23.6% vs. oral 19.8%, absolute risk difference of 3.8%, 95% (95% *CI* − 0.07 to 7.7%; *p* = 0.055) (Fig. 1). Assessing independently, severe desaturation events were not associated with nasal TI (nasal 23.0% vs. oral 18.8%, *p* = 0. 051). However, nasal TI was associated with lower occurrence of severe TIAE (nasal 2.2% vs. oral 3.8%, absolute risk difference of − 1.6%, 95% *CI* − 3.1 to − 0.02%, *p* = 0. 047) (Table 5).

**Table 5:** propensity score-matched analysis: absolute risk difference with 95% confidence interval in the primary and secondary outcomes

**Outcomes**	Nasal TI (*n* = 869)	Oral TI (*n* = 869)	**Absolute risk difference (95% CI and *p*-value)**
**Primary outcome** (severe desaturation and/or severe TIAE)	205/869 (23.6%)	172/869 (19.8%)	3.8% (− 0.07 to 7.7%, *p* = 0.055)
Severe desaturation (SpO_2_ decline > 20% from baseline)^a^	194/843 (23.0%)	154/817 (18.8%)	4.2% (− 0.02 to 7.9%, *p* = 0.051)
Severe TIAEs	19/869 (2.2%)	33/869 (3.8%)	− 1.6% (− 3.1 to − 0.02%, *p* = 0.047)
Any TIAEs	73/869 (8.4%)	85/869 (9.8%)	− 1.4% (− 4.1 to 1.4%, *p* = 0.337)
Multiple attempts	67/869 (7.7%)	86/869 (9.9%)	− 2.2% (− 4.8 to 0.4%; *p* = 0.101)
First attempt success	627/869 (72.2%)	621/869 (71.5%)	0.7% (− 0.04 to 0.05%;* p* = 0.759)

*CI *Confidence interval, *TI *Tracheal intubation, *TIAE *Tracheal intubation-associated events. Please refer to the “Materials and methods” section of the paper

^a^Note that desaturation data were not available for all TIs due to non-detectable SpO_2_ values

95% confidence interval and *p*-value are from bootstrap resampling

### Secondary outcomes

In the univariate analyses, any TIAEs were reported in 8.5% of nasal TI and 13.9% of oral TI (*p* < 0.001). However, in the PS-matched analysis, nasal TI was not associated with TIAEs (absolute risk difference − 1.4%, 95% *CI*: − 4.1% to 1.4%, *p* = 0.337). Multiple TI attempts (greater than two attempts) were observed in 7.7% of nasal TI and 10.5% of oral TI (*p* = 0.009). In the PS-matched analysis, the nasal route was not associated with multiple attempts (absolute risk difference − 2.2%, 95% *CI*: − 4.8 to 0.4%; *p* = 0.101). First attempt success rate was 72.1% of nasal TI versus 69.2% of oral TI, *p* = 0.072. In the PS-matched analysis, the nasal route was not associated with first attempt success (absolute risk difference 0.7%, 95% *CI*: − 3.7 to 5.1%, *p* = 0.759).

## Discussion

The aim of this study was to evaluate the association between primary nasal TI, severe desaturation, and TIAEs in a large and international prospective registry of TIs across PICUs. The occurrence of the primary outcome, either severe desaturation and/or severe TIAE, was similar in both groups in the univariate analysis, yet the severe TIAE was less common in the nasal TI group, and severe desaturation was more common in the nasal TI group. After adjusting for the imbalance in patient, provider, and practice characteristics with a multivariable logistic regression and in a PS-matched analysis, we did not observe a significant association of nasal TI with severe oxygen desaturation and/or severe TIAEs.

In our study, there was a significant difference in patient, provider, and practice characteristics related to children undergoing nasal versus oral TI. Infants with cardiac conditions were more prevalent in the nasal TI group, for instance. This is in concordance with the prior literature, where patients receiving nasal TI were mostly children under 2 years old (88.1%), with a cardiac disease (82.2%) [8]. In our study, nasal TI was also associated with procedural indication for TI. More attending physicians and subspecialists performed nasal TI. This may be explained by the fact that nasal TI procedure may require more airway experiences and technical skills, as it may be more challenging technically [14].

Among 22,741 primary TIs, fewer than 4% were by the nasal route. Our results are consistent with a recent retrospective cohort study of 121 PICUs in the USA, which reported that nasal TI was used in a minority of PICUs, and a similar small proportion (5.6%) of all 12,088 TIs were nasal TIs [8]. Of note, this study included academic and nonacademic medical centers, while the overwhelming majority of our NEAR4KIDS TI data were from academic centers.

We speculate that the choice of intubation route (i.e., nasal vs. oral) is determined by the physician’s experience and the clinical context, such as the patient’s physiological tolerance to intubation because duration of the TI procedure may be longer in the patient undergoing nasal TI. In a study by comparing nasal and oral TI on neonatal mannequins by inexperienced providers, longer time spent for the intubation procedure was reported in the nasal group (85 s in nasal TI vs. 48 s in oral group, *p* < 0.001). Lenclen et al. showed that the success rate for intubation with a duration less than 30 s was higher for the oral TI group (100% vs. 66% in nasal TI, *p* < 0.001) [14]. In the study by Abdelbaser et al., the median time needed for the intubation was significantly longer with nasal TI (31.5 s) compared to the oral group (16.0 s) (*p* < 0.001) [9]. Some may also consider that nasal TI is the preferred route for prolonged intubation in critically ill children, to improve tube stability and comfort, and to decrease unplanned extubation^10^. Christian et al. reported that nasal TI may be associated with lower occurrence of unplanned extubation compared with the oral TI group (0.9% vs. 2.9%, *p* < 0.001)^10^. Of prior literature report, no statistically significant difference in sinusitis and VAP between children with nasal TI and oral TI was found [8, 15]. Nasal intubation (vs. oral) at 24 h of endotracheal tube is associated with increased duration of invasive mechanical ventilation in children with bronchiolitis [16].

In our study, there were no difference in the severe TIAEs and/or severe oxygen desaturation in the nasal TI compared with oral group. In a randomized controlled trial of nasal TI versus oral TI evaluating post-extubation airway obstruction, complications of peri-intubation desaturation and bradycardia and more than one intubation attempt were comparable in both groups [15]. In a recent randomized controlled trial of nasal versus oral TI in infants and neonates who underwent a cardiac surgery, the change in S_p_O_2_ from baseline during intubation (3.4% vs. 3.2%, *p* = 0.826) and more than one intubation attempt were similar between the nasal TI and oral group [9]. Another study by Orlowski et al. also described similar rate of major complications occurring in children who had nasal versus oral TI (11% vs. 10%) [17]. Finally, in a Cochrane review of nasal versus oral TI for mechanical ventilation of newborn infants, the intubation failure rate was greater in the nasal TI compared with the oral TI, indicating the former procedure may be more difficult in this age group [18]. However, these studies did not report other peri-intubation adverse TIAEs or severe desaturation. The uniqueness of our study is the throughout evaluation of peri-intubation events, highlighting the importance of this prospectively collected data.

This study has several limitations. Our study was unable to report the duration of the TI procedure. This data point would require direct observation or video recording of TI procedure. Our study was also unable to address the outcomes related to mechanical ventilation with nasal endotracheal tube in place. These outcomes can include the occurrence of sinusitis, ventilator-acquired pneumonia (VAP), and unplanned extubation. In our study, the two groups of nasal versus oral TI were markedly unbalanced, in terms of prevalence as well as in patient, provider, and practice characteristics. We attempted to account for this, utilizing multivariate logistic regression and PS analysis, but we cannot exclude residual confounding factors. Although prospectively collected, an underreporting bias for TIAEs and desaturation may exist. In addition, detailed information regarding diagnosis and severity of illness was not recorded and may be of influence in the choice of route of TI. The type of unit (exclusively PICU versus mixed PICU and CICU) of PICUs included may limit the generalization of the study findings.

## Conclusion

In this large international prospective cohort, children receiving primary nasal TI did not have higher risk of severe peri-intubation desaturation and or severe TIAEs compared with those receiving oral TI. Nasal TI was infrequently used and associated with substantial differences in patient, provider, and practice. A prospective interventional multicenter trial is warranted to address the potential selection bias and to confirm the safety of nasal TI.

## Data Availability

The datasets used and/or analyzed during the current study are available from the corresponding author on reasonable request, providing approval by the Ethics Committee of the CHU Sainte‐Justine Research Center.
